# Effects of interleukin‐6 receptor blockade on allergen‐induced airway responses in mild asthmatics

**DOI:** 10.1002/cti2.1044

**Published:** 2019-06-14

**Authors:** Joana A Revez, Lisa M Bain, Rick M Watson, Michelle Towers, Tina Collins, Kieran J Killian, Paul M O'Byrne, Gail M Gauvreau, John W Upham, Manuel AR Ferreira

**Affiliations:** ^1^ QIMR Berghofer Medical Research Institute Brisbane QLD Australia; ^2^ Division of Respirology Department of Medicine McMaster University Hamilton ON Canada; ^3^ Diamantina Institute University of Queensland Brisbane QLD Australia

**Keywords:** actemra, allergy, GWAS, IL6, IL6R, rs2228145, trans‐signalling

## Abstract

**Background:**

Interleukin (IL)‐6 signalling has been implicated in allergic asthma by animal, genetic association and clinical studies. In this study, we tested the hypothesis that tocilizumab (TCZ), a human monoclonal antibody that blocks IL‐6 signalling, can prevent the development of allergen‐induced bronchoconstriction in humans.

**Methods:**

We performed a randomised, double‐blind, placebo‐controlled study, with eligible participants completing two allergen inhalation challenge tests, conducted before and after treatment with a single dose of TCZ or placebo. The primary efficacy endpoint was the magnitude of the late asthmatic response recorded between 3 and 7 after allergen challenge. The secondary efficacy endpoint was the early asthmatic response, measured 20 min to 2 h after allergen challenge.

**Results:**

A total of 66 patients enrolled between September 2014 and August 2017, when the trial was stopped for futility based on results from an interim analysis. Eleven patients fulfilled all eligibility criteria assessed at baseline and were subsequently randomised to the TCZ (*n *=* *6) or placebo (*n *=* *5) groups. Both the primary and secondary efficacy endpoints were not significantly different between the two groups. Five patients reported adverse events (AEs), three in the TCZ group (11 AEs) and two in the placebo group (four AEs). Only one AE was TCZ‐related (mild neutropenia), and there were no serious AEs. Significant treatment effects were observed for serum levels of C‐reactive protein, IL‐6 and soluble IL‐6R levels.

**Conclusion:**

In a small proof‐of‐concept clinical trial, we found no evidence that a single dose of tocilizumab was able to prevent allergen‐induced bronchoconstriction. (Trial registered in the Australian New Zealand Clinical Trials Registry, number ACTRN12614000123640).

## Introduction

Interleukin‐6 (IL‐6) was first described as a stimulatory factor released by T cells to induce antibody production by B cells.^1^ It was subsequently shown to be produced by other immune cells, such as macrophages^2^ and granulocytes,^3^ as well as non‐immune cells, including bronchial epithelial cells.^4^ Upon its release, typically triggered by cellular stress or damage, IL‐6 exerts its biological actions through two main pathways, termed classic signalling and trans‐signalling.

IL‐6 classic signalling involves binding of IL‐6 to the membrane‐bound IL‐6 receptor (mIL‐6R), which is expressed by a restricted group of cells, including some immune cells,^5^, ^6^, ^7^, ^8^, ^9^ airway epithelial cells^10^ and hepatocytes.^11^ IL‐6 binding to mIL‐6R triggers the dimerisation of the signal transducer gp130, which activates the JAK/STAT and the Ras/MAPK intracellular pathways.^12^ Activation of these pathways causes the nuclear translocation of transcription factors, such as STAT3, that regulate the expression of IL‐6 responsive genes.^13^ IL‐6 trans‐signalling is similar in that the same intracellular pathways are activated, but through a different mechanism. Briefly, IL‐6 binds to a soluble version of IL‐6R (sIL‐6R), which is produced via at least three processes: proteolytic shedding of mIL‐6R^14^ by endogenous (e.g. ADAM17) or microbial^15^ proteases; differential splicing of exon 9 of *IL6R*; and/or release of microvesicle‐associated IL‐6R.^16^ The IL‐6‐sIL‐6R complex is then recognised by gp130, which leads to IL‐6‐dependent activation of cells that do not express mIL‐6R. Because gp130 is ubiquitously expressed, IL‐6 trans‐signalling widens the range of cell types that respond to IL‐6. A third IL‐6 signalling mode was described recently, termed IL‐6 cluster signalling, which involves presentation of mIL‐6R‐bound IL‐6 by dendritic cells to gp130‐expressing T cells.^17^

Numerous studies have suggested a role for IL‐6 signalling in asthma pathophysiology, some of these reviewed previously.^18^, ^19^, ^20^ In clinical studies, IL‐6 levels have been reported to be elevated in asthmatics, both systemically and in the airways.^21^, ^22^, ^23^ In turn, high IL‐6 levels have been associated with reduced lung function^24^, ^25^, ^26^ and more severe disease symptoms.^24^, ^27^ Allergen (but not methacholine) challenge has also been shown to increase serum levels of both IL‐6^21^ and sIL‐6R.^28^, ^29^ These clinical associations are in line with results from functional studies that support a broadly pro‐inflammatory role of IL‐6 in asthma. For example, IL‐6 has been shown to promote (1) the differentiation of T helper (Th) 2 over Th1^30^, ^31^ and Th17 over Treg cells^32^, ^33^; (2) mucus production^34^; (3) immune cell recruitment, activation, proliferation and survival^35^, ^36^, ^37^; (4) obesity‐associated^38^ and LPS‐induced^39^ inflammation. Most of these pro‐inflammatory effects of IL‐6 have been suggested to be mediated by the trans‐signalling pathway.^38^, ^39^, ^40^, ^41^ Exceptions include, for example, the generation of pathogenic Th17 cells^17^, ^33^ and mucus production,^8^ which appear to be dependent on IL‐6 cluster and/or classic signalling. Although broadly pro‐inflammatory, IL‐6 can also mediate anti‐inflammatory effects, including inhibition of dendritic cell maturation^42^, ^43^; control of bacterial infections,^44^ likely mediated by classic signalling^45^; and mucosal protection from, or repair after, epithelial damage,^46^, ^47^, ^48^, ^49^ likely though trans‐signalling.^50^

A role for IL‐6 signalling on asthma pathophysiology is also supported by two parallel observations from human genetic association studies. The first was the discovery that a non‐synonymous variant (rs2228145:A > C) in exon 9 of *IL6R* 51 explains about 50% of the variation in serum levels of sIL‐6R.^52^, ^53^, ^54^, ^55^, ^56^ For each inherited copy of the rs2228145:C allele – which has a frequency of 39%, 38% and 13% in populations of European, East Asian and African ancestry,^57^ respectively – serum sIL‐6R levels increase by about 1200 pg mL^−1^.^56^ At least two mechanisms are likely to underlie this association. First, sIL‐6R can be directly produced from a transcript isoform that lacks exon 9, which partly encodes for the transmembrane domain.^58^ The rs2228145:C allele is associated with higher mRNA^55^, ^59^ and protein^60^ levels of this spliced isoform (DS‐sIL‐6R). However, each rs2228145:C allele increases DS‐sIL‐6R protein levels by about 100 pg mL^−1,^ 60 and so this effect explains only a small amount of the variation in steady‐state sIL‐6R levels, as previously highlighted.^60^, ^61^ Second, and perhaps more importantly, rs2228145 is thought to affect mIL‐6R shedding. This SNP encodes amino acid 358 (Asp > Ala) of IL‐6R, which is located in the extracellular stalk region and coincides precisely with the cleavage site used by ADAM17 to shed mIL‐6R.^62^, ^63^ Ferreira *et al*.^55^ noted that rs2228145:C was associated with substantially lower (~25% decrease per allele) mIL‐6R protein expression in primary immune cell types, despite no significant association with RNA levels of the full‐length *IL6R* transcript (based on *n *=* *88). This suggested that rs2228145:C (358Ala) strongly promotes ADAM17‐dependent shedding of mIL‐6R. Direct evidence for this has been reported in neutrophils,^64^ with ambiguous results for mononuclear cells.^60^, ^64^ Of note, subsequent larger studies of gene expression (e.g. *n* = 4467) found that rs2228145:C is in fact associated with lower overall *IL6R* transcription levels,^56^, ^65^ unlike observed in the smaller study by Ferreira *et al*.^55^ In summary, there are unambiguous associations between rs2228145:C and higher sIL‐6R (including DS‐sIL‐6R) in serum, lower mIL‐6R in immune cells and lower overall *IL6R* transcription, although the underlying molecular and cellular mechanisms are not yet fully elucidated.

In parallel, we found that the rs2228145:C allele was associated with a 1.09‐fold higher risk of asthma in individuals of European descent,^66^ an observation that has since been replicated in the UK Biobank study.^64^, ^67^ A similar association (odds ratio [OR] = 1.08) was also reported for atopic dermatitis (AD or eczema),^68^ with a stronger effect (OR = 1.22) observed for the persistent form of AD.^69^ Recently, we showed that rs2228145:C occurs at the same frequency in cases that suffer from asthma, hay fever or AD, therefore confirming its effect on the risk of multiple allergic diseases.^70^ Lastly, there is also evidence that rs2228145:C is associated with more severe disease symptoms and decreased lung function in patients with asthma,^71^, ^72^ but not with the risk of chronic obstructive pulmonary disease.^64^ In contrast to a predisposing effect on allergic disease, rs2228145:C is associated with decreased risk of coronary heart disease (OR = 0.95)^73^ – notably aortic aneurysms, atherosclerosis and myocardial infarction^74^ – rheumatoid arthritis (RA; OR = 0.93)^75^ and ankylosing spondylitis (OR = 0.88).^76^

The observed genetic associations between rs2228145 and allergic, cardiovascular and autoimmune diseases suggest that drugs that target the IL‐6 signalling pathways might help treat these conditions. Currently, at least eight such drugs are approved or in clinical development: three IL‐6R antagonists (tocilizumab, Roche; sarilumab, Regeneron; and vobarilizumab, Ablynx); three IL‐6 antagonists (siltuximab, Janssen; sirukumab, Janssen; and SA‐237, Chugai); and one IL‐6/sIL‐6R complex antagonist (olamkicept, Conaris). Of these, tocilizumab and sarilumab, both of which block mIL‐6R and sIL‐6R, are widely used to treat RA. Results from human genetic association studies suggest that the efficacy of these two drugs in RA might be largely due to inhibition of IL‐6 classic signalling and not due to inhibition of trans‐signalling. This is because the effect of the drug and of the disease‐protective allele (rs2228145:C) matches for IL‐6 classic signalling (inhibited by both) but not for trans‐ (inhibited by drug, promoted by allele) signalling. Consistent with this possibility, IL‐6 classic signalling was shown to be obligate and sufficient for the induction of systemic disease in a murine model of human arthritis.^77^ In contrast, for asthma and other allergic diseases, the disease‐protective allele is rs2228145:A, which inhibits IL‐6 trans‐signalling but promotes classic signalling. Based on this observation, we suggest that the inhibition of IL‐6 classic signalling *per se*, although potentially beneficial to attenuate local allergic immune responses,^78^ on balance is unlikely to be a successful therapeutic approach for allergic diseases. Instead, overall drug efficacy is likely to require inhibition of IL‐6 trans‐signalling, consistent with results from mouse studies.^8^, ^29^

Given the prediction from human genetic association studies that blockade of IL‐6 classic signalling could have an opposing effect on asthma symptoms when compared to blockade of trans‐signalling (aggravate and attenuate, respectively), it is not clear what effect should be expected from drugs that block both pathways, such as tocilizumab or sarilumab. Using mouse models of allergic asthma, we found that an IL‐6R mAb that blocks both pathways had a protective effect on allergen‐induced airway inflammation only when the experimental model used resulted in increased levels of sIL‐6R in the airways and so that was likely to involve activation of IL‐6 trans‐signalling^8^, ^29^. When that was not the case, dual receptor blockade resulted in worse airway inflammation when compared to control mice. Therefore, drugs such as tocilizumab or sarilumab might potentially have a beneficial therapeutic effect in subsets of patients with airway inflammation that involves activation of IL‐6 trans‐signalling. Interestingly, monthly treatment with tocilizumab, which has a half‐life of 13 days at the 8 mg kg^−1^ dose,^79^ was found to decrease clinical activity of AD in three patients treated for up to 12 months.^44^ This was the first indication in humans that inhibition of both mIL‐6R and sIL‐6R could be helpful to treat allergic diseases.

In this study, we performed a proof‐of‐concept clinical trial to test the hypothesis that a drug that blocks both IL‐6 classic signalling and trans‐signalling can be used to prevent allergen‐induced asthma exacerbations. Specifically, we conducted a randomised, double‐blind, placebo‐controlled phase 2 trial, with eligible participants completing two allergen inhalation challenge tests, conducted before and after treatment with a single dose of tocilizumab or placebo. To enrich the trial sample for asthmatics with high sIL‐6R levels, which promotes IL‐6 trans‐signalling, only individuals with rs2228145:AC or CC genotype were eligible to complete the trial. The primary efficacy endpoint was the magnitude of the late asthmatic response recorded after allergen challenge, a clinically relevant outcome in proof‐of‐concept studies.^80^

## Results

### Study population

**Figure 1:**
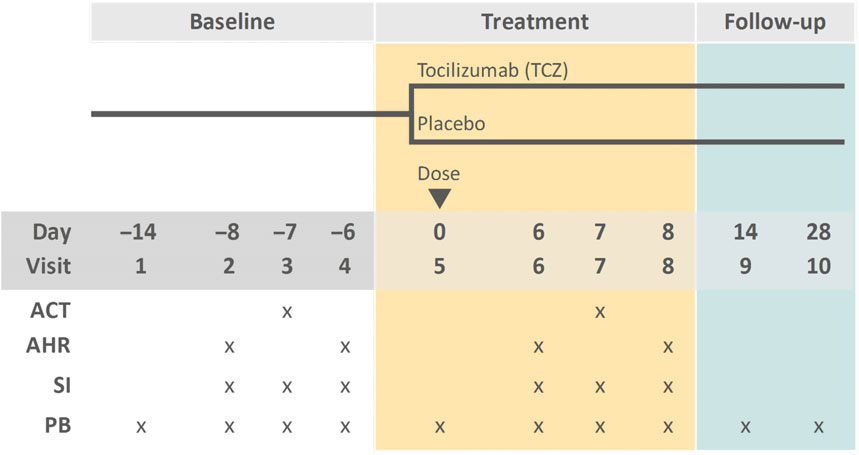
Study design. Arrow indicates the day on which tocilizumab or placebo was administered. Crosses indicate the days on which each procedure was performed. ACT: allergen challenge test. AHR: airway hyperresponsiveness test. SI: sputum induction. PB: peripheral blood collection.

A total of 66 patients enrolled between 29 September 2014 and 9 August 2017 (Supplementary table 1 and Supplementary figures 1 and 2), when the trial was stopped for futility based on results from the interim analysis (see Methods). Of these, 11 patients fulfilled all eligibility criteria assessed at baseline (visits 1 to 4) and so were randomised to receive a single dose of tocilizumab (TCZ; *n *=* *6) or placebo (*n *=* *5) at visit 5 (Figure 1). Demographic and clinical characteristics of patients at baseline were comparable between the two groups (Table 1). All 11 patients completed the subsequent five clinical visits, during which efficacy and safety endpoints were measured.

**Table 1:** Demographics and clinical characteristics of the 11 patients at baseline

Characteristics	Placebo	Tocilizumab
*N*	5	6
Mean age, years (SD)	29 (12.6)	35 (7.6)
N females (%)	4 (80)	4 (67)
Mean body mass index, kg m^−2^ (SD)	25.5 (3.8)	25 (4.0)
rs2228145 genotype, N with AC/N with CC	4/1	3/3
FEV_1_		
Mean, L (SD)	2.8 (0.4)	2.8 (0.8)
Mean percentage of the predicted value (SD)	92.2 (5.9)	90.7 (10.0)
Mean methacholine PC_20_, mg mL^−1^ (SD)		
24 h before allergen challenge	2.9 (2.1)	6.1 (5.03)
24 h after allergen challenge	1.1 (0.8)	2.2 (3.3)
Mean maximum percentage fall in FEV_1_ after allergen challenge (SD)		
During early asthmatic response (EAR)	30.9 (10.2)	31.7 (6.9)
During late asthmatic response (LAR)	25.4 (3.8)	21.9 (5.6)
Mean percentage of eosinophils in sputum (SD)		
24 h before allergen challenge	0.4 (1.0)	0.7 (1.3)
7 h after allergen challenge	3.8 (2.3)	28.1 (41.3)
24 h after allergen challenge	2.5 (3.4)	7.9 (7.3)
Mean percentage of neutrophils in sputum (SD)		
24 h before allergen challenge	15.2 (7.7)	45.8 (19.1)
7 h after allergen challenge	61.3 (14.1)	28.5 (20.3)
24 h after allergen challenge	45.8 (16.3)	56.1 (20.2)
Mean sIL‐6R levels 24 h before allergen challenge (SD)		
In sputum, pg mL^−1^	45.8 (42.2)	306.3 (261.8)
In serum, ng mL^−1^	120.2 (61.9)	151.3 (39.1)
Mean IL‐6 levels 24 h before allergen challenge (SD)		
In sputum, pg mL^−1^	5 (6.1)	33.5 (37.3)
In serum, pg mL^−1^	1.8 (3.2)	1.7 (4.1)

### Efficacy endpoints

The primary efficacy endpoint was the late asthmatic response (LAR), which was assessed based on two lung function outcomes recorded between 3 and 7 h after the allergen challenge. The first outcome, the maximum percentage fall in FEV_1_ (max%fall), was not significantly different between the TCZ and placebo groups (*P *=* *0.697, Table 2 and Supplementary table 2). There was also no significant treatment effect on the second LAR outcome analysed, the AUC of the per cent fall in FEV_1_ (AUC%fall; *P *=* *0.127, Table 2 and Supplementary table 2).

**Table 2:** Differences in efficacy endpoints between treatment groups

Endpoint	Outcome	*N*	ANCOVA P‐value	Estimated mean (SE)^a^		Estimated between‐group difference (SE)^a^
				Tocilizumab	Placebo	
Primary: LAR	max%fall	11	0.697	29.7 (6.0)	26.0 (8.0)	3.7 (9.1)
	AUC%fall	11	0.127	311.1 (11.3)	341.1 (15.1)	−30.0 (16.9)
Secondary: EAR	max%fall	11	0.741	40.0 (1.9)	40.9 (2.5)	−0.9 (2.6)
	AUC%fall	11	0.647	138.5 (7.6)	143.6 (9.6)	−5.1 (10.6)

AUC, area under the curve; AUC%fall, the AUC of the per cent fall in FEV1; EAR, early asthmatic response; LAR, late asthmatic response; Max%fall, maximum percentage fall in FEV1; SE, standard error.

a Estimated based on ANCOVA.

The secondary efficacy endpoint was the early asthmatic response (EAR), measured 20 min to 2 h after the post‐treatment allergen challenge. Both EAR outcomes analysed, max%fall and AUC%fall, were comparable between the TCZ and placebo groups (Table 2 and Supplementary table 2). Therefore, based on data from 11 patients who completed the trial, a single dose of TCZ did not significantly attenuate the allergen‐induced LAR or EAR.

### Safety endpoints

Five of the 11 patients reported adverse events (AEs; Supplementary table 3), three in the TCZ group (11 AEs) and two in the placebo group (four AEs). Only one AE was treatment‐related (mild neutropenia), and there were no serious AEs. Amongst the 55 patients who were not randomised to treatment, there were nine AEs and one SAE (Supplementary table 4).

Amongst the blood cell parameters measured (Supplementary table 5), significant differences between treatment groups at a nominal *P *<* *0.05 were observed for monocyte counts (lower in TCZ group at visit 6); neutrophil and total white blood cell counts (both lower in TCZ group at visit 7); and red blood cell counts (higher in TCZ group at visit 8). For blood biochemistry parameters (Supplementary table 6), a significant treatment effect was observed for insulin (higher in TCZ group at visit 7) and sodium (higher in TCZ group at visit 10) levels.

### Exploratory endpoints

There was no association between treatment group and the methacholine PC_20_, measured either 24 h prior (visit 6) or 24 h after (visit 8) the allergen challenge (Supplementary table 7). The relative number of eosinophils and neutrophils in sputum collected at visits 6 and 8 was also not significantly different between the TCZ and placebo groups (Supplementary table 8). Lastly, amongst the inflammatory mediators measured in blood and sputum samples (Supplementary table 9), significant treatment effects were observed in serum samples for CRP (lower in TCZ group at visits 7 and 8), IL‐6 (higher in TCZ group at visits 8 and 9) and sIL‐6R (higher in TCZ group at visits 6, 7 and 8).

## Discussion

The key finding from our randomised clinical trial was that patients with mild asthma who were pretreated with a single intravenous dose of TCZ experienced a reduction in lung function triggered by allergen inhalation that was comparable to that observed in patients pretreated with placebo. Possible explanations for the observed inability of TCZ to prevent allergen‐induced airway bronchoconstriction are discussed below, including inappropriate (1) drug concentration in the lung; (2) asthma exacerbation model used and patient group recruited; (3) drug type; and (4) drug target.

First, it is possible that the administration route (i.v.), drug dose (8 mg kg^−1^) and/or timing (on average 10 days prior to allergen challenge) of TCZ treatment did not achieve a drug concentration in the lung that was sufficient to effectively block IL‐6 trans‐signalling locally during the allergen‐induced asthmatic response. In healthy individuals, > 98% of sIL‐6R in serum is bound to TCZ between seven and 14 days after a single infusion at 2 mg kg^−1.^ 81 However, whether such high levels of sIL‐6R blockade in serum extend to the lung is unknown; this might not be the case if the concentration of TCZ in the lung is orders of magnitude lower than in serum, as shown for the anti‐IL‐5 antibody mepolizumab.^82^

Second, the provoked model of asthma that we used might not be the most effective approach to assess the efficacy of therapies that target non‐type 2 inflammatory pathways. Based on historical data,^83^ we expected that ~40% of patients tested using this model would develop airway inflammation during the LAR that was rich in both eosinophils and neutrophils, the inflammatory subtype that we^8^ and others^26^ have suggested is more likely to respond to TCZ treatment. However, at 7 h postchallenge, only one of the 11 patients was found to have mixed‐granulocytic sputum, with an additional two patients having this subtype at 24 h postchallenge. That is, most patients developed eosinophilic inflammation, which our previous work indicated was unlikely to be attenuated by TCZ.^8^ Because of funding constraints, it was not feasible in our trial to selectively recruit patients with mixed‐granulocytic sputum after allergen challenge, but this should be considered in future studies of IL‐6 signalling inhibitors in asthma. Another group of asthmatics not well represented in our study – and that has been suggested to potentially benefit from inhibition of IL‐6 signalling – consists of those with systemic inflammation, as defined by high serum levels of IL‐6.^84^

Third, a potential limitation of TCZ when considered for the treatment of asthma or other allergic diseases is that it blocks both mIL‐6R and sIL‐6R,^85^ that is, it inhibits both IL‐6 classic signalling and trans‐signalling. Findings from human genetic association studies (see Introduction) indicate that blocking mIL‐6R is likely to exacerbate asthma symptoms, because the rs2228145:C allele that promotes mIL‐6R shedding (i.e. that inhibits classic signalling, like TCZ) is associated with higher disease risk. For example, blockade of mIL‐6R might exacerbate asthma symptoms because it releases the inhibitory effect of IL‐6 on dendritic cell‐mediated T cell activation.^43^ We hypothesise that inhibition of this and other anti‐inflammatory effects of IL‐6 might partly explain why TCZ did not attenuate allergen‐induced bronchoconstriction in our clinical trial. To address this possibility, future clinical trials should consider drugs that block sIL‐6R but not mIL‐6R, such as olamkicept,^86^ which we^8^ and others^29^ have shown can significantly attenuate allergen‐induced airway inflammation in mice.

Lastly, it is possible that, despite support from human genetic association studies and experimental animal models of asthma, IL‐6R is not an appropriate drug target to prevent or treat allergen‐induced asthma exacerbations. For example, genetic association findings – specifically, the observation that rs2228145:C is associated with both higher risk of allergic diseases (asthma, hay fever and eczema) and higher sIL‐6R levels – might be explained by a major role of IL‐6 trans‐signalling during the development of atopy (i.e. allergic sensitisation) and not during allergen‐induced exacerbations in atopic individuals. There is, however, some evidence against this possibility; rs2228145:C is associated with worse lung function in asthmatics,^71^, ^72^ a finding that we found is supported by results from the UK Biobank study (*n *=* *31 471 Europeans with FEV_1_/FVC data; β = −0.026, SE = 0.007, *P *=* *4.8 × 10^−4^). Another level of complexity is that both mIL‐6R and sIL‐6R also bind the p28 subunit of IL‐27,^87^, ^88^ with the sIL‐6R/p28 complex having antiviral activity.^89^ IL‐27 has been implicated in the aetiology of allergic disease, both by functional^90^ and genetic association studies.^91^, ^92^ Therefore, a role for IL‐6R inhibition in IL‐27‐dependent allergic responses should also be considered in future studies.

In conclusion, in a small proof‐of‐concept clinical trial, we found no evidence that a single dose of tocilizumab was able to prevent allergen‐induced bronchoconstriction. We suggest that the design of future trials of IL‐6 signalling inhibitors in asthma should consider a drug dose, administration route and/or timing that results in adequate drug availability in the lung; selective recruitment of asthmatics with mixed‐granulocytic inflammation; and drugs that block sIL‐6R but not mIL‐6R.

## Methods

### Patients

Patients invited to participate in the trial were men or women who, in previous studies or in response to media appeals, reported being 18–65 years of age, non‐smokers, of general good health and with a history of asthma that did not require regular treatment with corticosteroids. Patients who completed the baseline phase of the trial (see below) and remained eligible to continue were the subset confirmed to have the rs2228145:AC or CC genotype, as well as mild, stable allergic asthma. The latter was determined based on a skin prick test to house dust mite (HDM; *Dermatophagoides farinae*), baseline spirometry and both methacholine and allergen inhalation challenge tests, as described in detail below.

### Study design

We performed a proof‐of‐concept, randomised, double‐blind, parallel‐group, placebo‐controlled study. Patients were recruited between September 2014 and August 2017 at three clinical sites: Q‐Pharm at the QIMR Berghofer Medical Research Institute (Brisbane, Australia); Princess Alexandra Hospital (PAH; Brisbane, Australia); and McMaster University (Hamilton, Canada). The study included 10 clinical visits, grouped into three phases: baseline, treatment and follow‐up (Figure 1).

#### Baseline phase (visits 1 to 4)

The following procedures were performed at visit 1 to assess eligibility criteria (Supplementary figure 1): informed consent; assessment of demographics and medical history; assessment of prior and concomitant medications (see Supplementary methods for details, including medications that were not allowed during the study, for example long‐acting B2 agonists, as well as inhaled or oral steroids); serum and urine pregnancy tests (women); physical examination; vital signs; spirometry; skin prick allergen challenge test with HDM; blood collection for assessment of serology (HIV, HBV, HVC), haematology and biochemistry parameters; electrocardiogram; chest X‐ray; urinalysis; DNA Sanger sequencing to assess genotype for rs2228145. Patients who satisfied all eligibility criteria assessed at this visit (Supplementary methods) – including a history of asthma, positive skin prick test to HDM, forced expiratory volume in 1 sec (FEV1) at baseline of 70% or more of the predicted value and rs2228145:AC or CC genotype – were invited to attend visit 2, which took place 6–20 days later. At visit 2, the following additional procedures were performed: skin prick allergen titration test, to identify the lowest concentration of HDM that caused a positive skin reaction; methacholine inhalation challenge test, to determine the presence of airway hyperresponsiveness (AHR); sputum induction and serum collection, for assessment of inflammatory mediators locally and systemically. Patients with a positive methacholine challenge test (defined below) and who satisfied all other eligibility criteria for this visit (Supplementary methods) were invited to attend visits 3 and 4, which took place 24 and 48 h later, respectively. At visit 3, patients performed the baseline (i.e. prior to drug intervention) allergen inhalation challenge test (described below) – the goal of this test was to elicit airway responses similar to those that follow natural allergen exposure. At visit 4, a methacholine challenge test was performed to determine whether the allergen challenge test from the previous day resulted in increased AHR, as typically occurs after a natural allergen‐induced asthma exacerbation. Patients with a positive allergen inhalation test (defined below) remained eligible to participate and so were invited to attend the treatment and follow‐up phases of the study.

#### Treatment phase (visits 5 to 8)

At visit 5, which took place 6–13 days after visit 4, patients were randomly assigned in a 1:1 ratio to receive a single intravenous infusion of tocilizumab at a concentration of 8 mg kg^−1^ (as recommended for the treatment of RA) or of placebo. The randomisation procedure used is described in the Supplementary methods. At visits 6 to 8, which took place 6–15 days after treatment (Supplementary figure 1), patients completed the same procedures as at visits 2 to 4, including a methacholine challenge test at visits 6 and 8, and the post‐treatment allergen inhalation challenge test at visit 7. Results from the pre‐ and post‐treatment procedures were compared to assess drug efficacy, as described in detail below.

#### Follow‐up phase (visits 9 and 10)

Two follow‐up visits were carried out to assess safety endpoints, specifically 14 (visit 9) and 28 (visit 10) days after treatment, in accordance with the published half‐life of tocilizumab (13 days for the 8 mg kg^−1^ dose).^79^ Considering the time interval allowed between visits (Supplementary figure 1), completing the full study could take between 34 and 70 days.

### Clinical procedures

#### Informed consent

Prior to enrolment, a signed patient information and consent form (PICF) was obtained for each potential participant. The PICF described the purpose of the study, the procedures to be performed, and the risks and benefits of participation. A clinical investigator conducted the informed consent discussion, including answering any questions about the study. Consent was voluntary and free from coercion.

#### Medical history

Information collected included current and previous illnesses, hospital admissions, known allergies, history of anaphylaxis, recent investigational drug treatments, history of tobacco and alcohol use.

#### Physical examination (including X‐ray)

The following parameters were measured: height, weight, heart rate, respiratory rate, blood pressure, temperature, examination of body systems, including a resting 12‐lead electrocardiogram, chest X‐ray and collection of a urine sample for analysis.

#### Lung function

The FEV_1_ and forced vital capacity (FVC) were measured with the patient sitting, using a MicroLab MK8 or nSpire spirometer that complies with 1995 ATS requirements, calibrated daily. Three largest acceptable spirograms were obtained, with the largest FEV_1_ and FVC retained; predicted values were calculated according to NHANES III.^93^

#### Skin prick challenge test

A drop of the HDM allergen extract (Jubliant HollisterStier LLC, WA, USA; Greer^®^, NC, USA), positive (histamine; Jubliant HollisterStier LLC, WA, USA) and negative (Jubliant HollisterStier LLC, WA, USA) controls was applied to the skin. The top layer of the epidermis was then punctured with a sterile lancet, one for each droplet. Excess antigen was absorbed with a tissue and the diameter of each wheal measured with a calliper in two (largest) perpendicular directions after 15 min. A reaction with HDM > 2 × 2 mm was considered a positive result, provided that the positive and negative controls elicited a reaction that was, respectively, larger and smaller than 2 × 2 mm.

#### Skin prick titration test

A control solution and twofold serial dilutions of the HDM allergen extract were applied to the skin 2.5 cm apart, from a concentration of 1:4 to 1:32 768. Skin reactivity was determined as described above. A single lancet was used for the HDM droplets, moving from lowest to highest concentration. The lowest concentration of allergen causing a skin wheal of at least 2 × 2 mm in size was recorded (termed “skin test endpoint”).

#### Methacholine inhalation challenge test

Patients with a baseline FEV_1_ that was ≥ 70% of the predicted value were eligible to perform a methacholine challenge test. As part of this test, first an aerosol containing a saline solution (0.9% sodium chloride) was inhaled using a dedicated Wright nebulizer (output of 0.13 mL min^−1^, calibrated at site initiation, then annually) connected to a 2‐way Hans Rudolf valve with mouthpiece, with the patient wearing a nose clip. Patients were instructed to breathe in a relaxed way by tidal breathing for 2 min. FEV_1_ was then measured 30 and 90 seconds after the end of the saline inhalation. Patients with an FEV_1_ at 90 seconds that was ≤ 80% of baseline or < 1 L did not continue with the methacholine challenge. If that was not the case, and if FEV_1_ at 90 seconds was the same or higher (i.e. ≥ 95%) than that at 30 seconds, patients proceeded with the methacholine challenge. If, in contrast, it was lower (i.e. < 95%) than that at 30 seconds, additional FEV_1_ measurements were obtained, first at 3 min postinhalation and then at 2‐min intervals thereafter, until the FEV_1_ started to rise. In this case, the patient continued with the methacholine challenge only if the lowest FEV_1_ recorded after inhalation was > 80% of baseline and > 1 L. The first methacholine aerosol had a concentration of 0.03 mg mL^−1^, followed by aerosols with doubling concentrations (up to 16 mg mL^−1^). Each aerosol was inhaled for 2 min, with a 5‐min interval between the end of one inhalation and the start of the next. FEV_1_ was measured 30 and 90 s after each aerosol. If the FEV_1_ at 90 seconds was the same or higher (i.e. ≥ 95%) than at 30 s, the next concentration of methacholine was administered. On the other hand, if it was lower (< 95%) than at 30 seconds, additional FEV_1_ measurements were obtained as described above. In this case, if, relative to the postsaline FEV_1_, the lowest postmethacholine FEV_1_ was (1) ≤ 80% or < 1 L, the test was stopped, and this was recorded as the methacholine provocation concentration that resulted in a 20% drop in FEV_1_ (i.e. methacholine PC_20_); (2) > 80% and ≤ 83%, the methacholine PC_20_ was extrapolated to minimise risk and discomfort to the patient; or (3) > 83%, the next concentration of methacholine was administered. This procedure was repeated until reaching the methacholine PC_20_ or the highest possible methacholine concentration (16 mg mL^−1^). If the methacholine PC_20_ was ≤ 16 mg mL^−1^, the test was considered positive.

#### Allergen inhalation challenge test

Patients with a baseline FEV_1_ that was ≥ 70% of the predicted value were eligible to perform an allergen challenge test. The starting concentration of the HDM allergen was 3–4 doubling concentrations below that predicted to cause a 20% fall in the FEV_1_ (allergen PC_20_). The predicted allergen PC_20_ was calculated using a formula described previously,^94^ which considers the methacholine PC_20_ and the skin test endpoint, both determined as described above. A solution containing 2 mL of the starting concentration of HDM was aerosolised with a dedicated Wright nebulizer and inhaled for 2 min through a 2‐way Hans Rudolph valve, as described above. Solutions containing doubling allergen concentrations were subsequently inhaled, with a 10‐min interval between the end of one inhalation and the start of the next. 10 min after the end of each inhalation, one technically acceptable FEV_1_ was measured. If, relative to the baseline FEV_1_, the postallergen FEV_1_ was (1) > 90%, then the next concentration was given; (2) > 80% and ≤ 90%, the FEV_1_ was measured again after a further 10 min – if that FEV_1_ was > 80% of the baseline FEV_1_, the next concentration or half concentration was given; (3) ≤ 80%, or at the discretion of the clinical investigator, the test was stopped. After the last allergen inhalation, FEV_1_ was measured at 11 time points postchallenge: 20, 30, 45, 60 and 90 min and then hourly at 2, 3, 4, 5, 6 and 7 h. If at any time point the FEV1 was < 50% of baseline, at the clinical investigator's discretion, the patient could be rescued with a bronchodilator and/or steroids. Otherwise, a bronchodilator was given after the 7 h time point. A positive allergen inhalation challenge test was defined by (1) a positive early asthmatic response (EAR), specifically a fall in FEV_1_ relative to baseline of at least 20% between 0 and 3 h after the last inhalation; and (2) a positive late asthmatic response (LAR), specifically a fall in FEV_1_ relative to baseline of at least 15% between 3 and 7 h after the last inhalation.

#### Sputum induction

Sputum induction was performed at six visits (Supplementary figure 1), immediately after the methacholine and allergen inhalation challenge tests. First, salbutamol was administered and three acceptable FEV_1_ measurements obtained after 10 min. If the best postsalbutamol FEV_1_ was ≥ 40% predicted and > 1 L, then a solution containing 15 mL of 3% hypertonic saline was inhaled for 7 min using a DeVilbiss or Universal ultrasonic nebulizer (DeVilbiss Healthcare), without the use of a nose clip. After the inhalation, the patient rinsed his/her mouth and was asked to cough sputum from the chest into a container. FEV_1_ was then measured; if, relative to the postsalbutamol FEV_1_, the postsaline FEV_1_ was (1) > 90%, then the next concentration of hypertonic saline (4% and then 5%) was given; (2) > 80% and ≤ 90%, the same concentration was used until the FEV_1_ returned to > 90% of the postsaline FEV_1_ (at which time the concentration was increased), the patient was exposed to a total of 21 min inhalation, or the FEV_1_ was ≤ 80% of the postsaline FEV_1_; (3) ≤ 80%, or if bothersome symptoms occurred, the inhalation was stopped and the patient treated with salbutamol. The sputum sample obtained was stored at 4°C and processed within 2 h.

#### Drug infusion

Tocilizumab (at a dose of 8 mg kg^−1^) and placebo (sterile saline solution) were acquired, prepared and labelled by the respective pharmacies at each clinical site. Both were administered at room temperature by controlled infusion into an arm vein over a 1‐h period at visit 5.

#### Blood collection

Up to 40 mL of peripheral blood were collected at each of the ten visits, after all other clinical procedures had been performed. The exception was visit 5, for which blood was collected prior to the drug infusion.

### Laboratory procedures

#### Sputum processing

Sputum was processed into three components: DTT‐free sputum supernatant, sputum supernatant with DTT and sputum cells. To obtain the first component, induced sputum was processed within 2 h from collection. Sputum plugs were separated from saliva and weighed. Sputum plugs were mixed in 1 × phosphate‐buffered saline (PBS), equivalent to 8 times the sputum plug weight, with a rotating mixer for 15 min. The sample was then centrifuged (790×*g*, 10 min, 4°C) with four times the volume of supernatant collected and stored at −80°C. To obtain the second component, sputolysin (Calbiochem‐Novabiochem Corp, San Diego, CA, USA) was added to the remaining sample to equal a final concentration of 0.1% DTT, which was gently mixed for 15 min with a rotating mixer. The sample was then filtered through 60 μm mesh into a preweighed tube. Cell counts and cell viability were determined with Trypan Blue (Sigma‐Aldrich, St. Louis). The sample was centrifuged (500×*g*, 10 min), and the supernatant was collected and stored at −80°C. To obtain the third component, the remaining cell pellet was resuspended to equate 1 × 10^6^ cells mL^−1^ and spun onto microscope slides (Shandon Cytospin, 500 rpm, 5 min), fixed in 90% ethanol for 10 min and stained with May‐Grünwald‐Giemsa for the differential cell counts. To obtain differential sputum cell counts, slides were scanned on the Aperio Scanscope XT at 40× magnification and viewed with Imagescope software (version 11.2.0.780). A total of 400 nonsquamous epithelial cells were counted, and percentages were calculated. Sputum samples with low viability (< 40%) were removed from the analysis of sputum cell counts.

#### Blood processing

Whole blood was separated via centrifugation (2000×*g*, 10 min, room temp). Plasma and buffy coat were collected from K3 EDTA tubes (Vacuette^®^), and serum was collected from Z serum clot activator tubes (Vacuette^®^). Plasma and serum were stored at −80°C, and DNA was extracted from the buffy coat. Whole blood was diluted (1:4) with 1 × PBS and layered onto Ficoll‐Paque PLUS, and the sample was centrifuged (400×*g*, 30 min, no brake). The upper plasma/platelet layer was discarded, and the mononuclear layer was transferred into a separate tube and washed with 1× PBS before cell counts and cell viability were determined by Trypan Blue (Sigma‐Aldrich, St. Louis). PBMCs were lysed with RLT buffer (RNeasy kit, QIAGEN, Germantown) and β‐mercaptoethanol (β‐ME), (10 μL β‐ME in 1000 μL RLT buffer) and stored at −80°C.

#### Measurement of cytokines in serum and sputum samples

Cytometric bead arrays (Becton Dickinson Biosciences, San Jose) were used to measure TNF (Flex Set C4 100Tst), IL‐13 (Flex Set E6 100Tst), IL‐8 (Flex Set A9 100Tst), IL‐6 (Flex Set A7 100Tst) and IL‐5 (Flex Set A6 100Tst). Enzyme‐linked immunosorbent assays were used to measure neutrophil elastase (BMS269; Invitrogen, Waltham; measured in sputum only), sIL‐6R (SR600; R&D Systems, Minneapolis) and CRP (R&D Systems, DCRP00; measured in serum only). Cytokine levels in sputum were determined using the DTT‐free supernatant samples.

#### DNA sequencing

DNA was extracted from buffy coats, and the DNA region surrounding SNP rs4129267 – which is a polymorphism in complete linkage disequilibrium (*r* ^2^ = 1, *D*′ = 1) with rs2228145 – amplified and analysed using Sanger Sequencing, at the Australian Genome Research Facility (accredited by the National Association of Testing Authorities, Australia). Genotype for rs4129267 (CC, CT or TT) was determined directly from the sequencing data, visualised with Chromas Lite version 2.1.1 software. Genotype for rs2228145 (AA, AC or CC) was imputed based on the observed rs4129267 genotype, given that rs2228145:A is always in phase with (i.e. on the same haplotype as) rs4129267:C. In some cases, to increase recruitment efficiency, potential eligible participants provided a 4 mL blood sample for rs4129267 genotyping prior to attending visit 1. Informed consent for this pre‐enrolment test was obtained in a separate PICF.

### Study endpoints

The primary endpoint was the late asthmatic response (LAR), as measured 3 to 7 h after the allergen challenge. Two outcome measures were used to evaluate the LAR: (1) the maximum percentage fall in FEV_1_ (max%fall), with larger values corresponding to worse airway function; and (2) the area under the curve (AUC) of the per cent fall in FEV_1_ (AUC%fall), with larger values corresponding to better airway function.

The secondary endpoints were as follows: (1) the early asthmatic response (EAR), as measured by the two outcomes described above between 20 min and 2 h after the allergen challenge; and (2) safety outcomes, namely frequency and severity of adverse events, vital signs, haematology and blood biochemistry results.

Exploratory endpoints were methacholine PC20; immune cell counts in sputum; levels of TNF, IL‐13, IL‐8, IL‐5 and neutrophil elastase, in blood and sputum.

### Statistical analysis

We compared post‐treatment outcomes for all endpoints between tocilizumab‐ and placebo‐treated patients using a repeated‐measures analysis of covariance (ANCOVA) in R version 3.2.2. Drug (tocilizumab vs. placebo) was the independent variable, while study site (three levels) and the baseline value for the outcome of interest were included as covariates in the linear model, which was: outcome ~ factor(site) + outcome_baseline + factor(drug). For laboratory outcomes measured after treatment at visits 6, 7, 8, 9 and 10, baseline values were those obtained before treatment at visits 2, 3, 4, 5 and 1, respectively. Unless otherwise noted, laboratory outcomes were log transformed, with the estimated marginal means reported after back‐transformation to the original scale. The R package emmeans (v1.3.1) was used to estimate the marginal mean and standard error (SE) of individual outcomes within each treatment group (given by emmeans(model, “drug”)), as well as between‐group differences or ratios (given by emmeans(model, pairwise ~ “drug”)). All *P*‐values reported in the results section correspond to the effect of drug treatment on the outcome of interest estimated in the ANCOVA. AUC was calculated using the R package Bolstad2.

### Target sample size and power

The target number of patients to complete the trial was 16, eight per treatment group. With this sample size, and using the ANCOVA analytical approach described above, the study had 80% power (α = 0.05, two‐sided test) to detect an absolute difference of 11.5% between placebo‐ and tocilizumab‐treated patients for the primary endpoint measured after treatment – specifically the max%fall during the LAR. This was determined using the approach described by Borm *et al.,* 95 and assuming that (1) the standard deviation (SD) of the LAR max%fall was 10% and (2) the correlation between the LAR max%fall measured in the same individuals at two time points was 0.6. These statistics were estimated based on results from allergen challenges performed in previous clinical trials,^83^ specifically pretreatment data for the LAR max%fall (mean = 23%) measured in 24 individuals tested with the same allergen twice in a 12‐month period. An absolute between‐group difference of 11.5% corresponds to a relative attenuation in LAR max%fall of 50% (i.e. 11.5%/23% = 0.5).

### Study oversight

The medical monitor was Professor Ian Yang (Prince Charles Hospital, Brisbane). The Data Safety and Monitoring Board (DSMB) was composed of the following: Professor Peter Sly (chair; Child Health Research Centre, University of Queensland, Brisbane); Professor Peter Nash (Department of Medicine, University of Queensland, Brisbane); Professor Daman Langguth (Wesley Hospital, Brisbane); and Dr Paul Griffin (Q‐Pharm, Brisbane). Adherence to Good Clinical Practice guidelines was monitored by Clinical Network Services (CNS) Pty Ltd.

All study procedures were approved by the ethics committees of the QIMR Berghofer Medical Research Institute (project P2025 and P2103), Metro South (HREC/14/QPAH/22 ‐ SSA/14/QPAH/216) and McMaster University (project 14‐790) and carried out according to the Declaration of Helsinki, the NHMRC National Statement on Ethical Conduct in Research Involving Humans (1999) and the Notes for Guidance on Good Clinical Practice (GCP) as adopted by the Australian Therapeutic Goods Administration (2000) (CPMP/ICH/135/95) and the GCP guidelines released by the International Council for Harmonisation of Technical Requirements for Pharmaceuticals for Human Use (ICH). All participants provided written informed consent before enrolling in the study. The trial was registered in the Australian New Zealand Clinical Trials Registry (ANZCTR), number ACTRN12614000123640.

### Interim analysis

In August 2017, after reviewing blinded results from 11 patients who had completed the ten clinical visits, the data safety and monitoring board (DSMB) requested an interim analysis of efficacy to assess futility. Based on the distribution of the primary endpoint (specifically, the max%fall recorded during the LAR) in the two treatment groups, the DSMB determined that continuing the trial to the target sample size (*n *=* *16) would not provide adequate power to detect a significant treatment effect, and so the trial was stopped for futility. Unblinded data for the 11 patients were then released to the investigators for detailed analyses, which are described above.
