# Supplementary figures and images for: Effects of interleukin‐6 receptor blockade on allergen‐induced airway responses in mild asthmatics
Source: Clin Transl Immunology. 2019 Jun 14;8(6):e1044. doi: 10.1002/cti2.1044 (PMC6566140; doi:10.1002/cti2.1044)

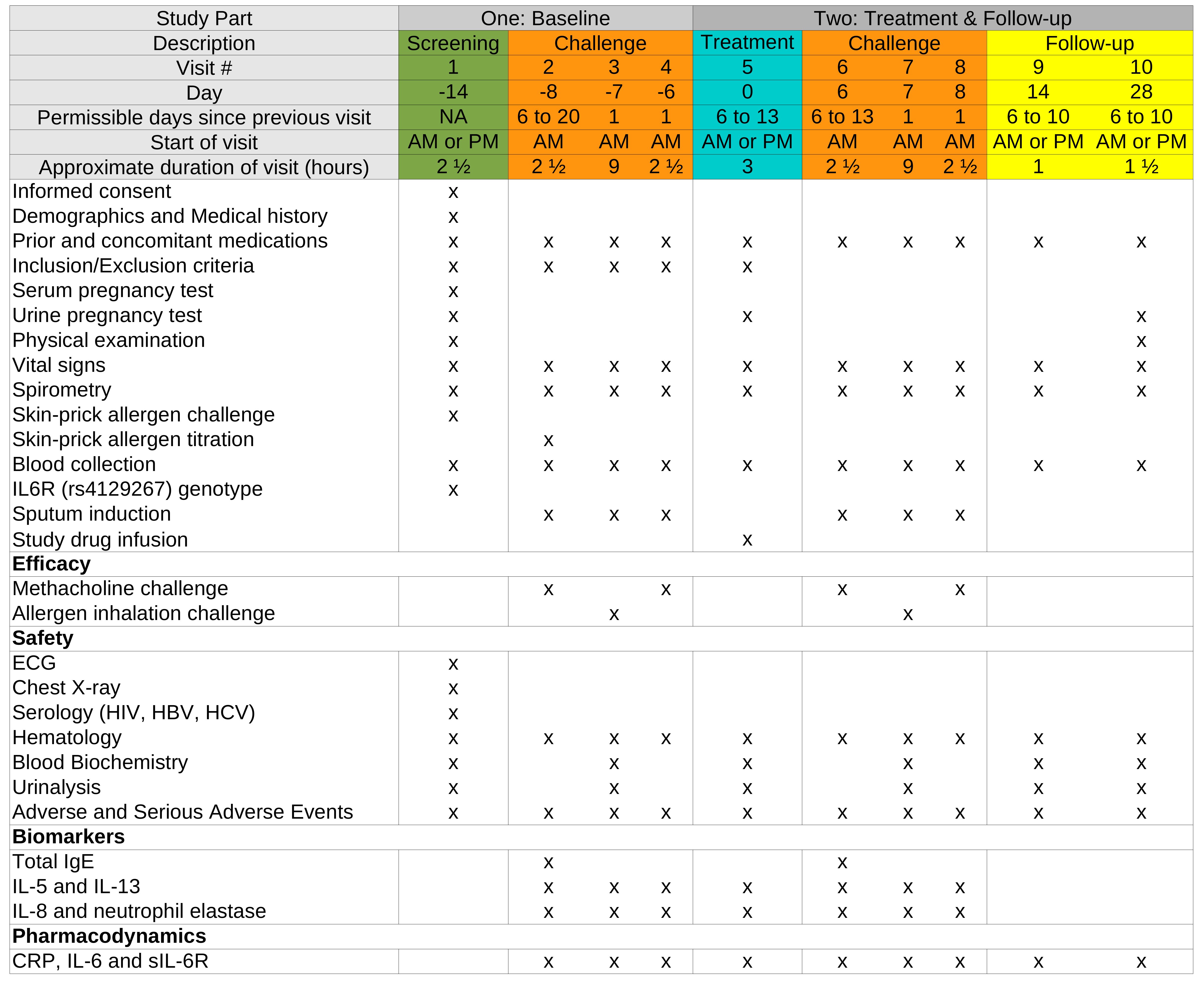

Supplement: Supplementary file 1 [file CTI2-8-e1044-s001.jpeg]

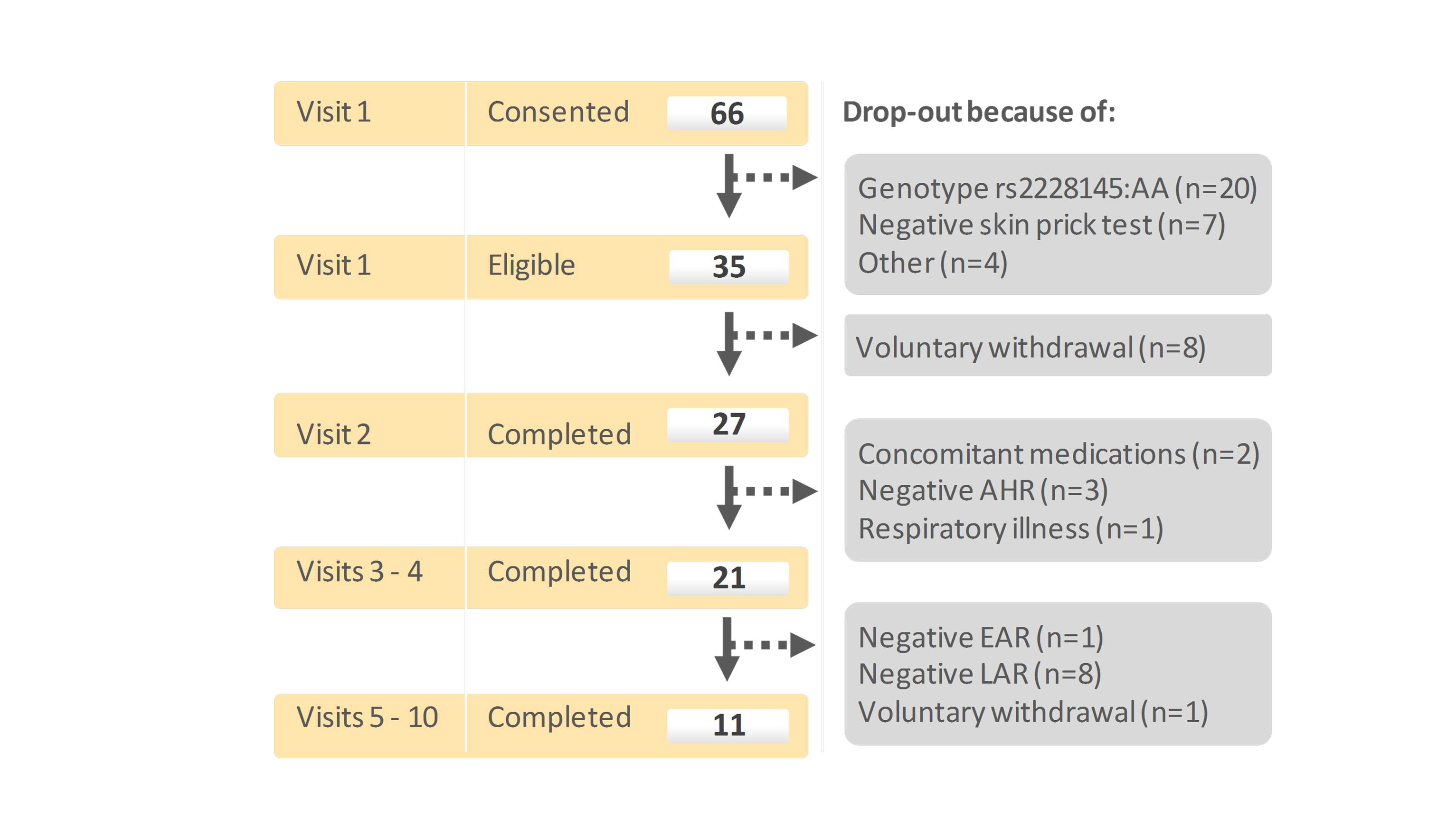

Supplement: Supplementary file 2 [file CTI2-8-e1044-s002.jpg]
