# Supplementary material for: Effects of interleukin‐6 receptor blockade on allergen‐induced airway responses in mild asthmatics
Source: Clin Transl Immunology. 2019 Jun 14;8(6):e1044. doi: 10.1002/cti2.1044 (PMC6566140; doi:10.1002/cti2.1044)
Supplement: Supplementary file 4 [file CTI2-8-e1044-s004.docx]

**SUPPLEMENTARY METHODS**

**Procedure used to assess drug washout requirements**

The following procedure was used to ensure that appropriate medication washout/withhold periods were satisfied prior to each visit.

List of specific medications to be screened:

| **Medication** | **Generic names** (not exhaustive) | **Brand names** |
| --- | --- | --- |
| Antibody therapy | | |
| Allergen immunotherapy | | |
| Immuno-suppressives | Methotrexate, mercaptopurine,  dactinomycin | |
| Inhaled steroids | Budesonide, fluticasone propionate, mometasone furoate, ciclesonide,  beclomethasone | Flixotide, Pulmicort, Alvesco, QVAR, Symbicort, Seretide |
| Systemic steroids | Cortisone, hydrocortisone, prednisone, betamethasone, dexamethasone | Normison, Predsolone, Predsone, Dexmethsone |
| Nasal/Topical steroids | Budesonide, fluticasone, monohydrate mometasone furoate | Budamax, Novasone, Zatamil, Elocon, |
| Theophylline | | Nuelin |
| Nedocromil or cromoglycate | | Intal, Tilade |
| Long-acting anticholinergics | Tiotropium bromide monohydrate | Spiriva |
| Anticholinergic | Ipratropium | Atrovent |
| Long-acting B2 agonists | Salmeterol or formoterol | Oxis, Serevent, Foradile |
| Short-acting B2 agonists | Salbutamol, fenoterol bromhydrate, terbutaline sulfate | Ventolin, Airomir, Asmol, Bricanyl |
| Leukotriene modifiers | Montelukast | Singulair |
| Long-acting antihistamines | Levocabastine , fexofenadine, cetirizine | Livostin, Fexal, Xergic, Telfast, Zilarex, Zyrtec, Alzena |
| Intermediate-acting antihistamines | Cyproheptadine, promethazine, loratadine | Periactin, Allereze, Lorano, Claratyne |
| Short-acting antihistamines | Promethazine |  |
| Salicylates | Acetylsalicylic acid | Aspirin |
| Other NSAIDs | Ibuprofen, naproxen, diclofenac sodium | Brufen, Inza, Naprosyn, Proxen, Crysanal, Phebra, Voltaren, Anaprox, Clonac, Fenac |
| Methylxanthines | Caffeine, theobromine | Coffee, guarana, tea (black and green), chocolate |
| Anticoagulants | Warfarin, heparin, enoxaparin, dalteparin | Coumadin, Marevan, Clexane, Fragmin |

Procedure:

1. At visits 1 to 10, record details (name, start/stop dates and dosages) for drugs used for any condition in the past 6 months (visit 1 only) or since the previous visit (visits 2 to 10).
2. At visit 1, establish if washout periods prior to study start are satisfied:

| **Medication** | **Washout prior to visit 1** |
| --- | --- |
| Antibody therapy | 5 months |
| Allergen immunotherapy | 4 months |
| Immuno-suppressives | 4 weeks |
| Inhaled steroids | 4 weeks from regular dosing |
| Systemic steroids | 4 weeks from regular dosing |
| Nasal/Topical steroids | 4 weeks from regular dosing |
| Theophylline | 4 weeks |
| Nedocromil or cromoglycate | 4 weeks |
| Long-acting anticholinergics | 2 weeks |
| Long-acting B2 agonists | 2 weeks |
| Leukotriene modifiers | 2 weeks |

*Was the subject on any of the therapies above during the times indicated?*

NO: continue. YES: ineligible.

1. At visit 1, inform subject of the drugs that may/may not be used during the study:
   1. The following drugs may be used during the study but require withholding prior to specified laboratory visits:
      - Nasal/Topical steroids (if used at a constant dose throughout the study and subject meets all study criteria while taking these medications), methylxanthines, short-acting β2 agonist (inhaled), anticholinergics, antihistamines, aspirin, other NSAID’s. Withholding times are indicated in the Table below and were listed in the appointment confirmation letter for each visit sent to the subject.
   2. The following drugs may not be used during the study; they require washout if subjects have been using or if acute treatment is required:
      - Steroids (inhaled, systemic), leukotriene modifiers, long- acting β2 agonist (inhaled), long-acting anticholinergics, antibody therapy, immunotherapy, immunosuppressives, theophylline, anticoagulants, nedocromil or cromoglycate .
2. At visits 1 to 10, establish if withholding periods for allowed drugs (section 3a above), or washout periods from acute dosing of drugs not allowed (section 3b above), are satisfied for each procedure:

| **Medication** | **Allowed during study?** | **Withholding or washout**  **prior to procedure** | **Procedure** | | | |
| --- | --- | --- | --- | --- | --- | --- |
|  |  |  | Spirometry | Skin-prick | Methacholine challenge | Allergen challenge |
| Leukotriene modifiers | No | 2 weeks | x | x | x | x |
| Long-acting B2 agonists | No | 2 weeks | x | x | x | x |
| Inhaled steroids | No | 1 week | x | x | x | x |
| Systemic steroids | No | 1 week | x | x | x | x |
| Long-acting antihistamines | Yes | 9 days |  | x |  | x |
| Salycylates | Yes | 7 days |  |  |  | x |
| Intermediate-acting antihistamines | Yes | 4 days |  | x |  | x |
| Other NSAIDs | Yes | 3 days |  |  |  | x |
| Short-acting antihistamines | Yes | 2 days |  | x |  | x |
| Nasal/Topical steroids | Yes | 12 hours |  |  | x | x |
| Short-acting B2 agonists | Yes | 8 hours | x |  |  |  |
| Methylxanthines | Yes | 4 hours | x |  |  |  |

*Considering the procedures relevant for a given visit, was the subject on any of the therapies above during the times indicated?*

NO: continue protocol.

If YES:

- For visits 1, 2, 3 or 5, attempt to reschedule visit, conditional on required washout and permissible days since previous visit (see Supplementary Figure 1). If not possible to reschedule, patient is ineligible to continue.
- For visits 4 and 8 (methacholine challenge 24h after allergen challenge), proceed with protocol if appropriate, recording details of acute treatment taken.
- For visit 6, attempt to reschedule visit, conditional on required washout and permissible days since visit 5. If not possible to reschedule, patient is ineligible to continue.
- For visit 7 (allergen challenge), attempt to reschedule visits 6 and 7, conditional on required washout and permissible days since visit 5. If not possible, patient is ineligible to continue.
- For visits 9 and 10, attempt to reschedule visit. A minimum of one follow-up visit should be carried out.

**Procedure used to assess eligibility criteria at visits 1 to 5**

Visit 1

1. **Assess outcomes of procedures “Informed Consent”, “Demographics & Medical history” and “Prior and concomitant medications”:**
   1. *Was the subject able to understand the study, the extent of his/her participation, agreed to participate and provided written informed consent using the study consent form approved by HREC?*

YES: continue. NO: ineligible.

- 1. *Is the subject between 18 and 65 years of age?*

YES: continue. NO: ineligible.

- 1. *Is the subject male OR female and not pregnant/not lactating/not actively seeking pregnancy?*

YES: continue. NO: ineligible.

- 1. *Is the subject using, or willing to use, adequate and effective contraception during the study period?*

YES: continue. NO: ineligible.

- 1. *Does the subject have a history of general good health; mild to moderate, stable, allergic asthma; and history of episodic wheeze and shortness of breath?*

YES: continue. NO: ineligible.

- 1. *Are any of the following present:*
     - A worsening of asthma or a respiratory tract infection within 6 weeks preceding study entry.
     - History of clinically significant hypotensive episodes or symptoms of fainting, dizziness, or lightheadedness.
     - History or symptoms of cardiovascular disease, particularly coronary artery disease, arrhythmias, hypertension, or congestive heart failure.
     - History or symptoms of significant neurologic disease, including transient ischemic attack (TIA), stroke, seizure disorder, or behavioral disturbances.
     - History of serious adverse reaction or hypersensitivity to any drug.
     - History or symptoms of clinically significant autoimmune disease.
     - History of clinically significant hematologic abnormality, including coagulopathy.
     - Participation in any other investigational drug treatment protocol within the preceding 30 days or 5 half lives of the drug.
     - Use of tobacco products of any kind currently or within the previous 12 months, or smoking history > 10 pack years.
     - Lung disease other than mild to moderate allergic asthma.
     - Concomitant disease or condition which could interfere with the conduct of the study, or for which the treatment might interfere with the conduct of the study, or which would, in the opinion of the investigator, pose an unacceptable risk to the subject in this study, including, but not limited to, cancer, alcoholism, drug dependency or abuse, or psychiatric disease.
     - Recent (less than 1 year) history of alcohol dependency
     - Contra-indications for TCZ are present, including:
  - Known hypersensitivity to any component of the product (recombinant humanised monoclonal antibody, polysorbate 80, sucrose, dibasic sodium phosphate dodecahydrate, monobasic sodium phosphate dihydrate ) or Chinese hamster ovary cell products.
  - A history of any reaction consistent with hypersensitivity to any component of the product or Chinese hamster ovary cell products.
  - Previous history of intestinal ulceration or diverticulitis.

If NO to all: continue. If YES to at least one: ineligible.

- 1. *Were drug withholding/washout periods prior to study start satisfied?*

YES: continue.

NO: ineligible.

- 1. *Were drug withholding/washout periods prior to study procedures for visit 1 satisfied?*

YES: continue.

NO: reschedule visit 1.

If subject remains eligible:

- - - Continue protocol for visit 1.

1. **Assess outcomes of procedures “Physical examination”, “Vital signs” and “Spirometry”:**
   1. *If female, is the urine pregnancy test negative?*

YES: continue. NO: ineligible.

- 1. *Is the subject in good health?*

YES: continue. NO: ineligible.

- 1. *Is the subject's FEV_1_ at baseline at least 70% of the predicted value?*

YES: continue. NO: ineligible.

- 1. *Is the chest X-ray normal?*

YES: continue. NO: ineligible.

- 1. *Is the electrocardiogram normal?*

YES: continue. NO: ineligible.

If subject remains eligible:

- - - Continue protocol for visit 1.

1. **Assess outcomes of procedure “Skin-prick allergen challenge”:**
2. *Was there a positive skin-prick test to the allergen D. farinae?*

YES: continue. NO: ineligible.

If subject remains eligible:

- - Continue protocol for visit 1.
  - Continue eligibility assessment at visit 2 (see below).

Visit 2

1. **Assess results from laboratory procedures conducted at visit 1:**
   1. *If female, is the serum pregnancy test negative?*

YES: continue. NO: ineligible.

- 1. *Are laboratory test results for hematology, chemistry, urinalysis and coagulation within normal range?*

YES: continue. NO: ineligible.

- 1. *Are laboratory test results for HIV, hepatitis B, HCV and EBV negative?*

YES: continue. NO: ineligible.

- 1. *Are baseline alanine aminotransferase and aspartate aminotransferase levels ≤1.5x ULN?*

YES: continue. NO: ineligible.

- 1. *Are neutrophil counts ≥2 x 10^9^/L and platelet counts ≥100 x 10^9^/L?*

YES: continue. NO: ineligible.

- 1. Was rs2228145 genotype AC or CC?

YES: continue. NO: ineligible.

If subject remains eligible:

- - - Continue protocol for visit 2.

1. **Assess outcome of “Concomitant medications” procedure:**
   1. *Were drug withholding/washout periods prior to study procedures for visit 2 satisfied?*

YES: continue.

NO: attempt to reschedule visit 2. If not possible, subject is ineligible.

If subject remains eligible:

- - - Continue protocol for visit 2.

1. **Assess results from “Methacholine Challenge” procedure:**
   1. *Was the subject's PC_20_ ≤ 16 mg/mL?*

YES: continue. NO: ineligible.

If subject is eligible:

- - Continue protocol for visit 2.
  - Continue eligibility assessment at visit 3 (see below).

Visit 3

1. **Assess outcome of “Concomitant medications” procedure:**
   1. *Were drug withholding/washout periods prior to study procedures for visit 3 satisfied?*

YES: continue.

NO: attempt to reschedule visits 2 and 3. If not possible, subject is ineligible.

If subject is eligible:

- - Continue protocol for visit 3.
  - Continue eligibility assessment at visit 4 (see below).

Visit 4

1. **Assess outcome from “Allergen Challenge” procedure:**
   1. *Did the subject develop a severe asthma reaction during or since allergen challenge?*

NO: continue. YES: ineligible.

1. **Assess results from “Screen for LAR” procedure:**
   1. *Did the subject develop both an EAR and LAR?*

YES: continue. NO: ineligible.

1. **Assess outcome of “Concomitant medications” procedure:**
   1. *Were drug withholding/washout periods prior to study procedures for visit 4 satisfied?*

YES: continue. NO: ineligible.

If subject remains eligible:

- - Continue protocol for visit 4.
  - Continue eligibility assessment at visit 5 (see below).

Visit 5

1. **Assess outcome of “Concomitant medications” procedure:**
   1. *Were drug withholding/washout periods prior to study procedures for visit 5 satisfied?*

YES: continue.

NO: attempt to reschedule visit 5. If not possible, subject is ineligible.

1. **If female, assess results from urine pregnancy test:**
   1. *Is the urine pregnancy test negative?*

YES: continue. NO: ineligible.

If subject remains eligible:

- - Continue protocol for visit 5, including randomization to treatment with tocilizumab or placebo.

**Procedure used for randomisation to treatment with tocilizumab or placebo**

The following block randomisation procedure was used prior to the start of the study at the PAH and Q-Pharm clinical sites to allocate tocilizumab or placebo to patients. Briefly, we: (1) assigned 16 drug kits to four blocks, each block containing four kits each; (2) randomly assigned tocilizumab or placebo at a 1:1 ratio to the four kits of each block, so that each block always contained two kits with tocilizumab and two kits with placebo, in a random order; and (3) randomly matched patients 1 to 4 with drug kits in block 1, patients 5 to 8 with drug kits in block 2, patients 9 to 12 with drug kits in block 3 and patients 13 to 16 with drug kits in block 4. The resulting randomisation table was held by the DSMB Secretariat throughout the duration of the study. Based on the randomisation table, two envelopes were created for each participant and sent to the respective pharmacy. Both envelopes contained the same information, namely the patient number, the block number, the kit number and the kit content. Envelope 1 was labelled with the project name, project number, patient number and with “Pharmacy Randomisation code” and “To be opened by trial pharmacist only to prepare the investigational drug”. Envelope 2 was labelled with the project name, project number, patient number and with “Emergency unblinding envelope” and “To be opened by investigator only in an emergency situation”. One to two days prior to visit 5, a study doctor completed a script requesting an investigational drug kit for the patient. The investigator then collected the investigational drug kit, together with envelope 2, which remained closed for the duration of the study. Envelope 1 was kept at the respective pharmacy until the conclusion of the study. A similar randomisation procedure was used at McMaster University.
